# RUNX2 promotes the suppression of osteoblast function and enhancement of osteoclast activity by multiple myeloma cells

**DOI:** 10.1007/s12032-023-01960-8

**Published:** 2023-03-10

**Authors:** Beihui Huang, Huixin Liu, Szehoi Chan, Junru Liu, Jingli Gu, Meilan Chen, Lifen Kuang, Xiaozhe Li, Xingding Zhang, Juan Li

**Affiliations:** 1 Department of Hematopathology, The First Affiliated Hospital, Sun Yat-Sen University, No. 58, Zhongshan 2Nd Road, Guangzhou, 510080 China; 2 Department of Pharmacology, School of Medicine, Molecular Cancer Research Center, Sun Yat-Sen University, No.66, Gongchang Road, Shenzhen, 518107 China

**Keywords:** RUNX2, Multiple myeloma, Bone destruction, Osteoblast, Osteoclast

## Abstract

RUNX2 is a transcription factor that participates in osteoblast differentiation and chondrocyte maturation and plays an important role in the invasion and metastasis of cancers. With the deepening of research, evidence has indicated the correlation between RUNX2 and bone destruction in cancers. However, the mechanisms underlying its role in multiple myeloma remain unclear. By observing the induction effects of conditioned medium from myeloma cells on preosteoblasts (MC3T3-E1) and preosteoclasts (RAW264.7) and constructing myeloma-bearing mice, we found that RUNX2 promotes bone destruction in multiple myeloma. In vitro, conditioned medium from RUNX2-overexpressing myeloma cells reduced osteoblast activity and increased osteoclast activity. In vivo, RUNX2 expression was positively correlated with bone loss in myeloma-bearing mice. These results suggest that therapeutic inhibition of RUNX2 may protect against bone destruction by maintaining the balance between osteoblast and osteoclast activity in multiple myeloma.

## Introduction

Multiple myeloma (MM) is an incurable dyscrasia with second-highest incidence in hematologic malignancy, which is attributed to the buildup of monoclonal plasma cells in the bone marrow [1, 2]. Approximately 230,000 patients are diagnosed with MM every 5 years [3]. Nearly 80% of new-diagnosed patients are detected with osteolytic lesions which contribute to the considerably elevated risk of death [4]. Attention to bone disease is a critical part of the therapeutic strategy for myeloma patients. Previous studies have demonstrated that the suppression of osteoblast function and enhancement of osteoclast activity plays significant role in the pathogenesis of myeloma bone disease [5]. Various pathways participate in the bone remodeling process, such as the receptor activator of nuclear factor (NF)-κB (RANK)/RANK ligand (RANKL) signaling pathway, Notch signaling pathway and tumor necrosis factor (TNF) superfamily, which are implicated in increased osteoclast activity, as well as the Wingless and integration-1 (WNT) signaling pathway, Dick-kopf-1 (DKK1) pathway and sclerostin pathway, which are implicated in decreased osteoblast activity [6–8].

Runt-related transcription factor 2 (RUNX2) is a transcription factor from Runx family which is consist of RUNX1, RUNX2, and RUNX3, and RUNX2 is vital for bone development [9]. It is expressed in multipotent mesenchymal cells, osteoblast-lineage cells, and chondrocytes. RUNX2 is conducive to osteoblast differentiation and chondrocyte maturation by directly regulating Ihh and Sp7 expression and canonical Wnt signaling [10]. In addition, recent studies have confirmed that RUNX2 is closely related to the proliferation and invasion of cancers, such as osteosarcoma, breast cancer, prostate cancer, gastric cancer, lung cancer, melanoma, and colorectal cancer [11–13]. RUNX2 is overexpressed in the above malignant tissues and stimulates the expression of metastatic marker genes such as vascular endothelial growth factor (VEGF), metalloproteinase 2 (MMP-2), osteopontin (OPN), and parathyroid hormone-related peptide (PTHrP), which ultimately leads to higher TNM stage and a poor prognosis in patients [14–16].

As an essential factor involved in bone development, RUNX2 exhibits an adverse effect on bone metastasis in multiple cancers. Previous studies have shown that RUNX2 contributes to the progression of breast and prostate cancer-related osteolytic bone destructions by inducing TGFβ signaling and the CTGF-RUNX2-RANKL axis [17–19]. New evidence has revealed that RUNX2 participates in the regulation of CD44-intracellular domain to the transcription of matrix metalloprotease-9 (MMP-9), which leads to enhanced bone resorption [16]. In MM, RUNX2 derived from myeloma cells facilitates tumor development through Akt/*β*-catenin/survivin signaling and upregulates the expression of multiple metastasis-related genes, which might be responsible for myeloma bone resorption [20]. Further study revealed that blocking RUNX2 in myeloma cells suppressed osteolytic lesions in vivo [21]. However, the detailed mechanism of the interaction between osteoclasts and osteoblasts in RUNX2-induced bone destruction, especially osteoblast activity transformation, remains unclear.

In this study, we show that increased expression of RUNX2 is responsible for bone destruction in MM. We demonstrate that RUNX2 promotes the suppression of osteoblast activity and enhancement of osteoclast activity by multiple myeloma cells using in vitro and in vivo approaches. Importantly, our data provide evidence that therapeutic inhibition of RUNX2 may protect against bone destruction by maintaining the balance between osteoblast and osteoclast activity in MM.

## Results

### RUNX2 is a poor prognostic factor in terms of survival and relapse in patients with MM

**Fig. 1:**
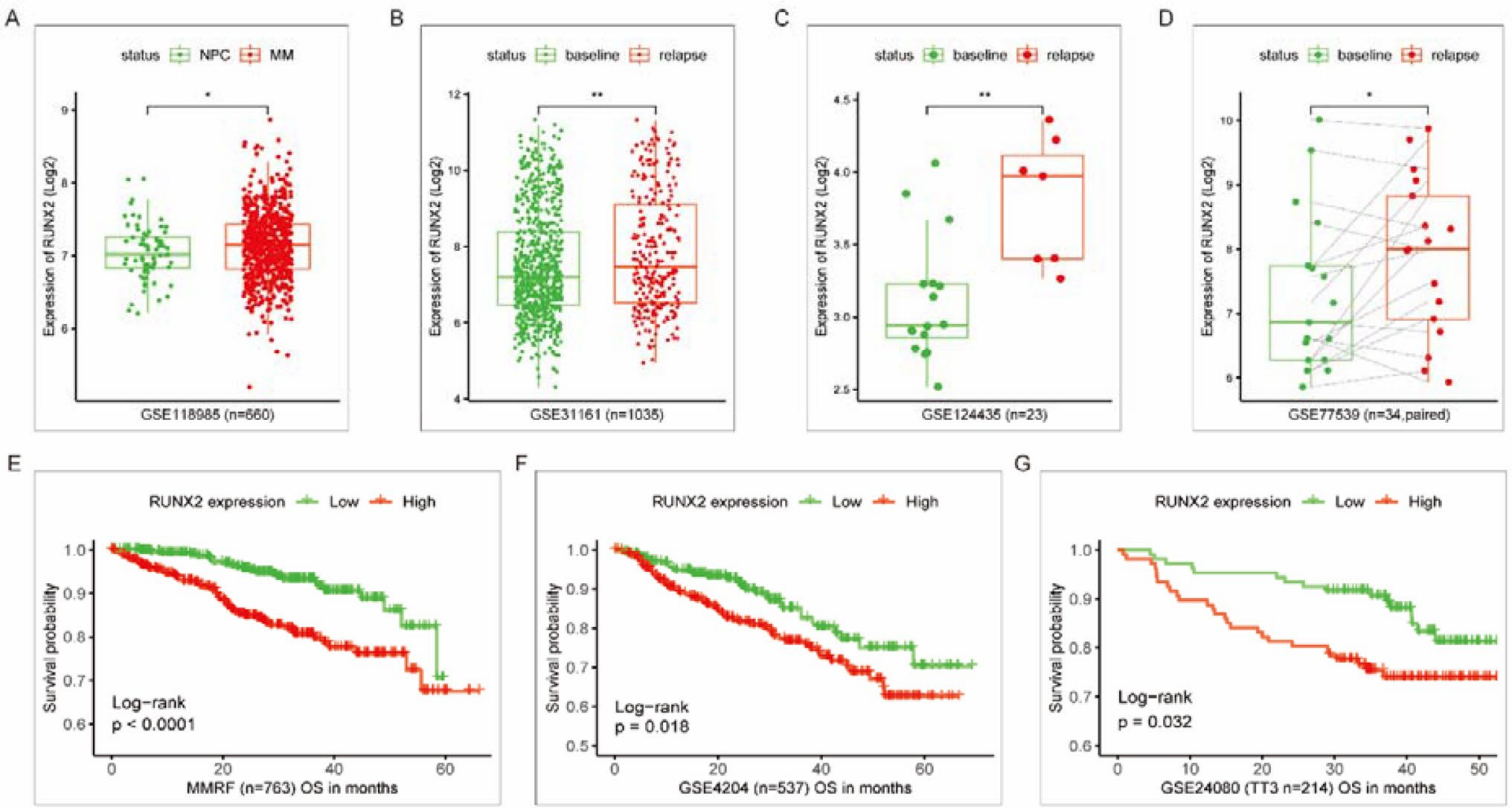
Analysis of RUNX2 expression data and survival data from a public database. **A** Comparison of RUNX2 expression in MM plasma cells and normal plasma cells. **B–D** Comparison of RUNX2 expression in paired and unpaired baseline and relapsed MM samples. **E, F** Survival curves of MM patients based on high and low RUNX2 expression levels

Data from a public database were collected to analyze the expression of RUNX2 in MM patients and its correlation with survival. Compared with that in normal plasma cells from healthy donors, the expression of RUNX2 was elevated in MM patient plasma cells (Fig. 1A). Relapsed MM exhibited a higher level of RUNX2 than newly diagnosed MM at baseline (Fig. 1B–D). Kaplan–Meier survival curves were generated for the high and low RUNX2 expression groups, which confirmed that high RUNX2 expression was correlated with shorter overall survival time (Fig. 1E, F). The results indicate that RUNX2 is a poor prognostic factor in terms of survival and relapse in MM.

### RUNX2 expression positively correlates with bone destruction in patients with MM

**Fig. 2:**
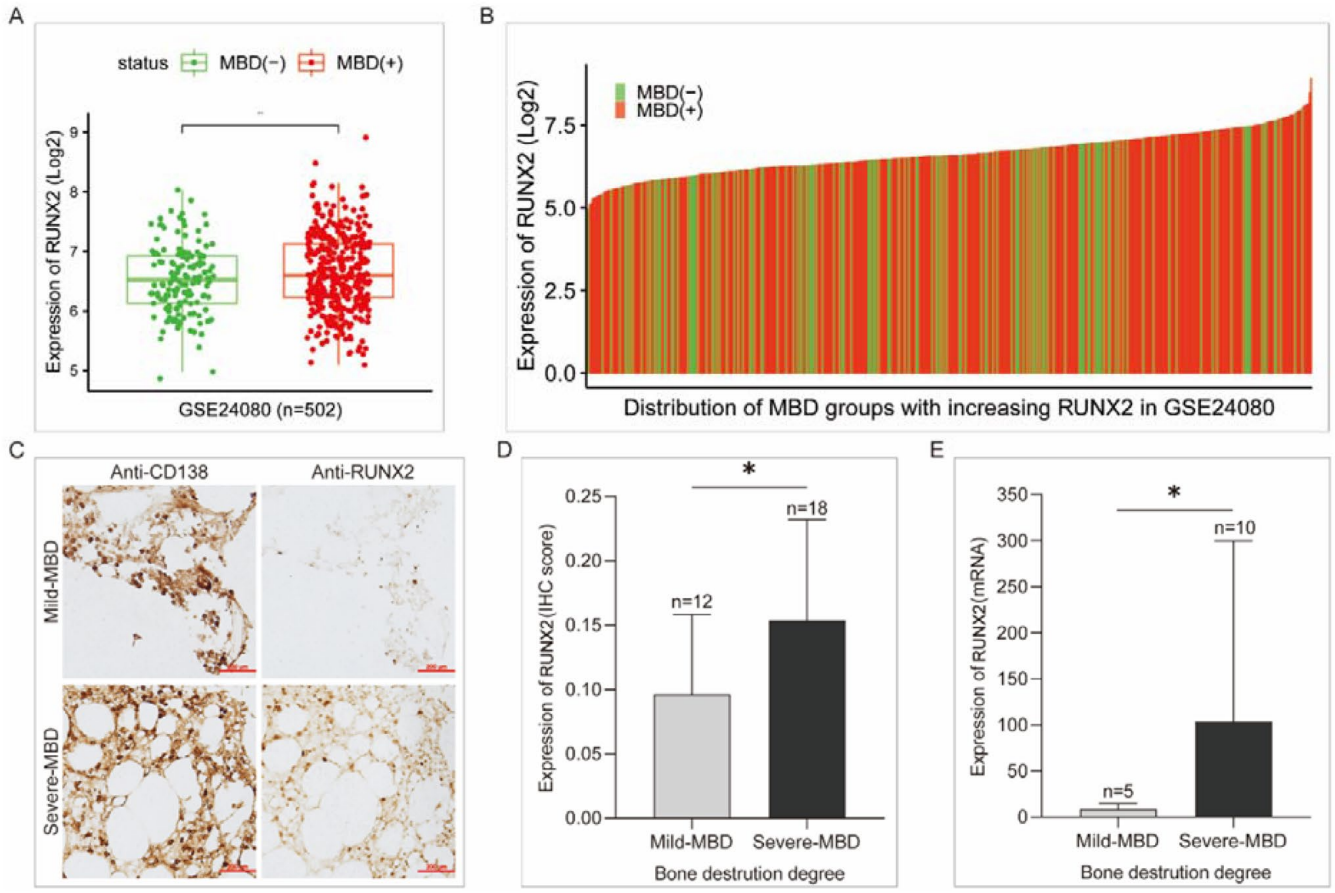
Correlation of RUNX2 expression and bone destruction in MM. **A, B** Comparison and distribution of RUNX2 expression between MM patients with and without MBD. **C** IHC staining of RUNX2 in bone biopsies from MM patients with mild or severe MBD based on similar tumor cell densities indicated by CD138. **D, E** Statistical analysis of RUNX2 expression between the mild and severe MBD groups based on IHC and qPCR results

To confirm the correlation of RUNX2 expression and myeloma bone destruction (MBD), we compared RUNX2 expression between samples with different degrees of MBD. MM patients with MBD showed higher RUNX2 levels than those without MBD (Fig. 2A). Subsequently, we collected 30 bone marrow biopsy sections and 18 fresh bone marrow fluid samples from newly diagnosed MM patients and divided them into mild and severe MBD groups according to the median level of bone destruction. Immunohistochemical (IHC) staining of bone biopsies was assessed on the background of CD138-positive staining to allow comparison of samples with similar tumor cell densities (Fig. 2C). According to both the IHC staining score and qPCR analysis, RUNX2 expression was higher in the severe MBD group than in the mild MBD group (Fig. 2D, E). These results indicate that the degree of MBD is related to the expression level of RUNX2 in MM, and the aggravation of bone destruction is accompanied by an increase in RUNX2 expression, confirming that RUNX2 is a molecule that promotes the development of MBD.

### Upregulation of RUNX2 promotes the suppression of myeloma osteoblast activity

**Fig. 3:**
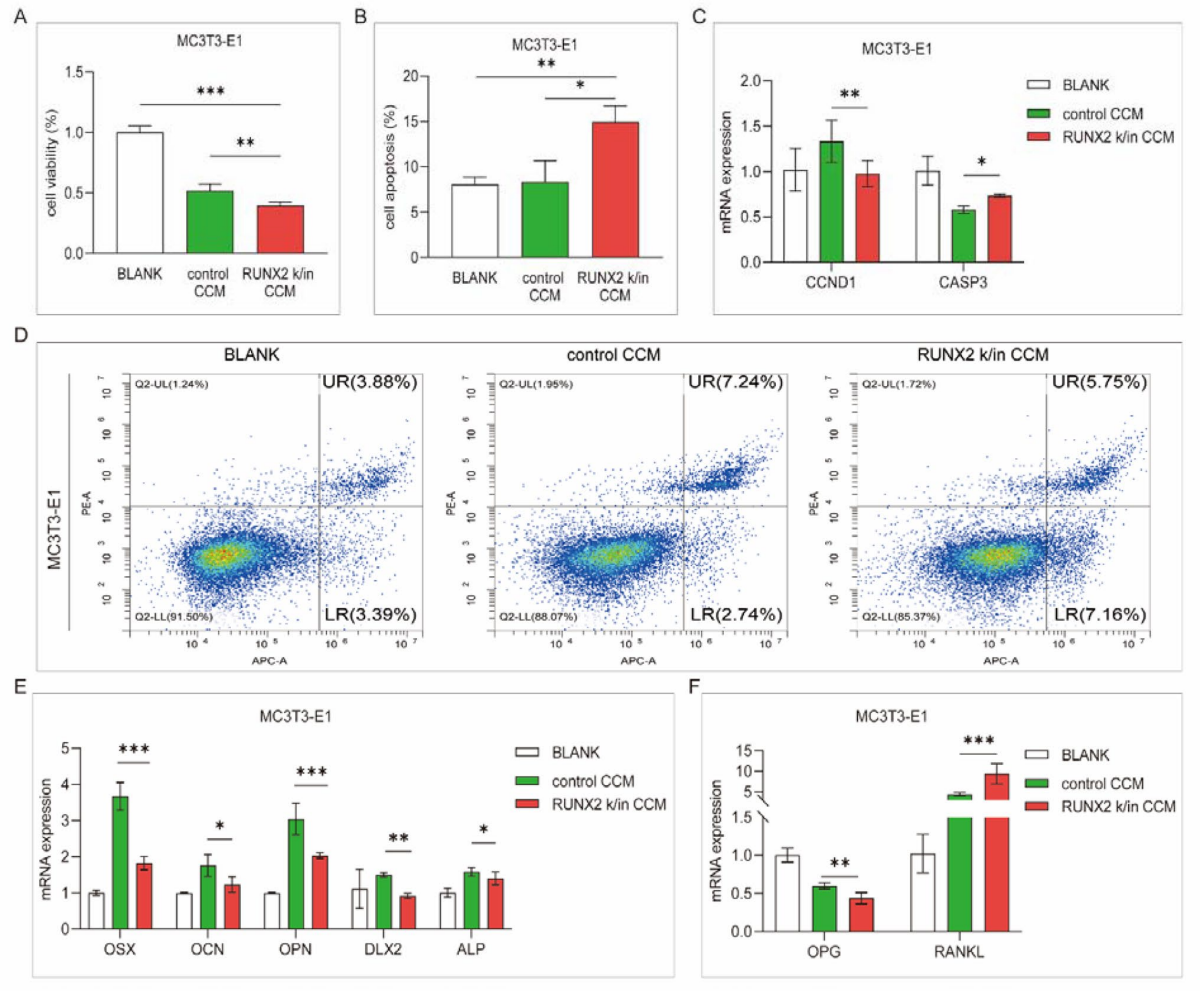
Analysis of osteoblast activity after incubation with CCM. MC3T3-E1 cells were incubated with serum-free medium (BLANK), control CCM or RUNX2 k/in CCM for 24 h.** A** Cell proliferation was detected with CCK-8 assay. **B** Cell apoptosis was detected by flow cytometry with an apoptosis kit. **C** CCND1 and CASP3 expression was detected by qPCR. **D** Representative images of flow cytometry apoptosis analysis. **E** The expression of differentiation-regulating genes in osteoblasts was detected by qPCR. **F** OPG and RANKL expression was detected by qPCR

Control and RUNX2 knock-in (k/in) 5TGM1 cells were incubated with serum-free medium for 24 h, and the supernatant was collected as conditioned medium. Subsequently, MC3T3-E1 cells were cocultured with concentrated conditioned medium (CCM) and assessed after 24 h. The RUNX2 k/in CCM group exhibited a higher cell viability rate and a lower apoptosis rate than the control group (Fig. 3A, B, *p*  < 0.05). The expression of CCND1, which is correlated with cell proliferation, was decreased, and that of CASP3, which is involved in cell apoptosis, was increased in the RUNX2 k/in the CCM group (Fig. 3C). To evaluate the differentiation of osteoblasts, we further detected the expression of differentiation-regulating genes, including OSX, OCN, OPN, DLX2, and ALP, which decreased after RUNX2 k/in CCM incubation (Fig. 3E). Communication with osteoclasts is another important aspect of osteoblast biological function in bone remodeling [22]. Osteoblasts induce osteoclast differentiation and development by secreting nuclear factor-kappa B ligand (RANKL), and this effect is attenuated by the secretion of osteoprotegerin (OPG), which is a soluble RANKL decoy receptor [23]. Compared with control CCM incubation, RUNX2 k/in CCM incubation contributed to the increased expression of RANKL and decreased expression of OPG in MC3T3-E1 cells (Fig. 3F). The results suggest that myeloma cells with upregulated RUNX2 not only inhibit the proliferation and differentiation of osteoblasts but also promote the differentiation of osteoblasts to osteoclasts, which indirectly suppresses osteoclast activity.

### Upregulated RUNX2 facilitates the promotion of osteoclast activity by myeloma cells

**Fig. 4:**
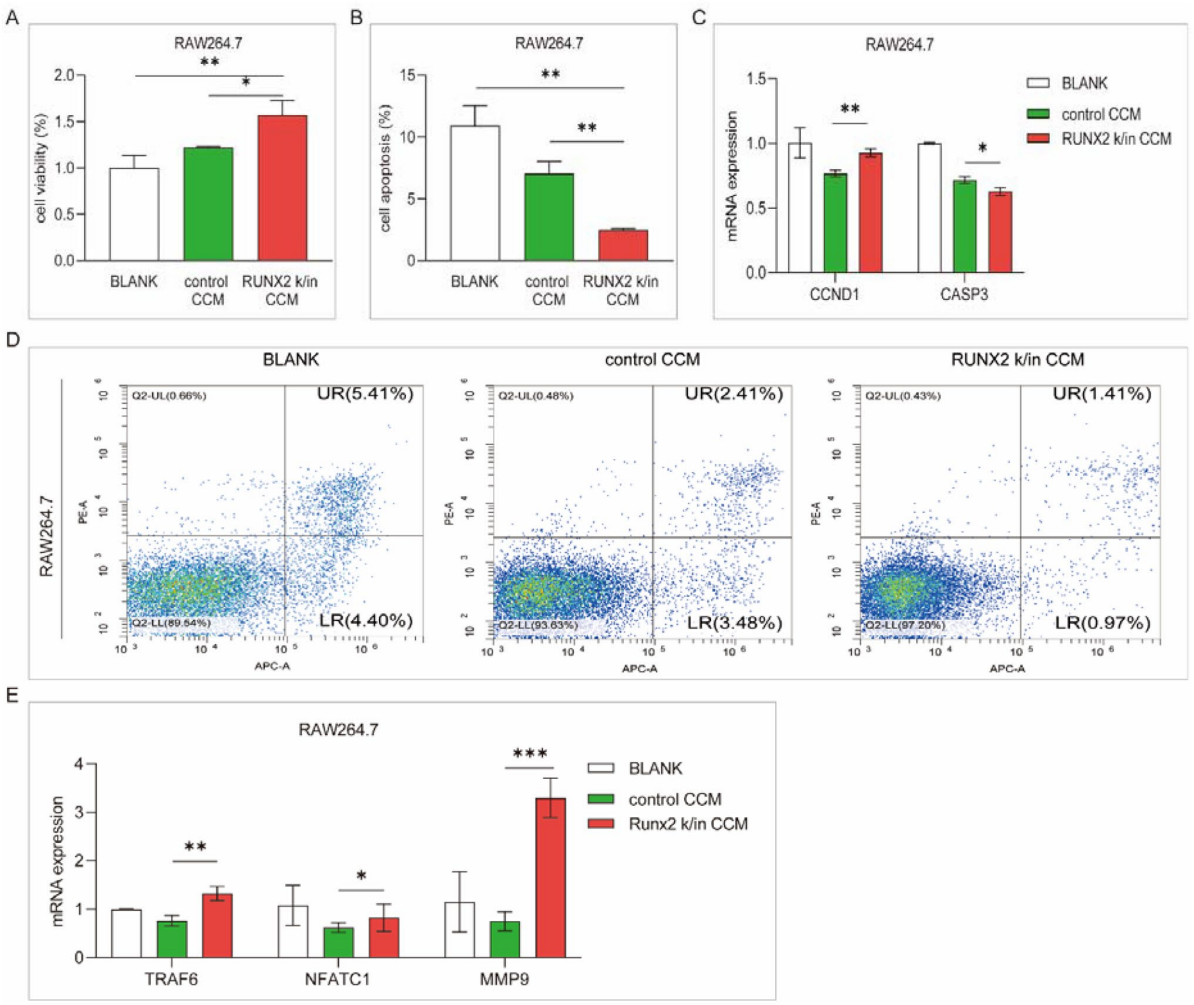
Analysis of osteoclast activity after incubation with CCM. RAW264.7 cells were incubated with serum-free medium (BLANK), control CCM, or RUNX2 k/in CCM for 24 h. **A** Cell proliferation was detected with a CCK-8 assay. **B** Cell apoptosis was detected by flow cytometry with an apoptosis kit. **C** CCND1 and CASP3 expressions were detected by qPCR.** D** Representative images of flow cytometry apoptosis analysis. **E** The expression of differentiation-regulating genes in osteoblasts was detected by qPCR

RAW264.7 cells were cocultured with control CCM and RUNX2 k/in CCM for 24 h. Compared with control CCM, RUNX2 k/in CCM increased the viability and suppressed the apoptosis of RAW264.7 cells (Fig. 4A, B). The expression of CCND1 was increased and the expression of CASP3 was decreased in the RUNX2 k/in CCM group (Fig. 4C). Representative genes related to osteoclast differentiation, such as TRAF6, NFATC1, and MMP-9, were significantly upregulated after RUNX2 k/in CCM incubation (Fig. 4E). These results support that RUNX2 promotes osteoclast proliferation and differentiation mediated by MM cells.

### LCN2 and M-CSF are downstream of RUNX2

**Fig. 5:**
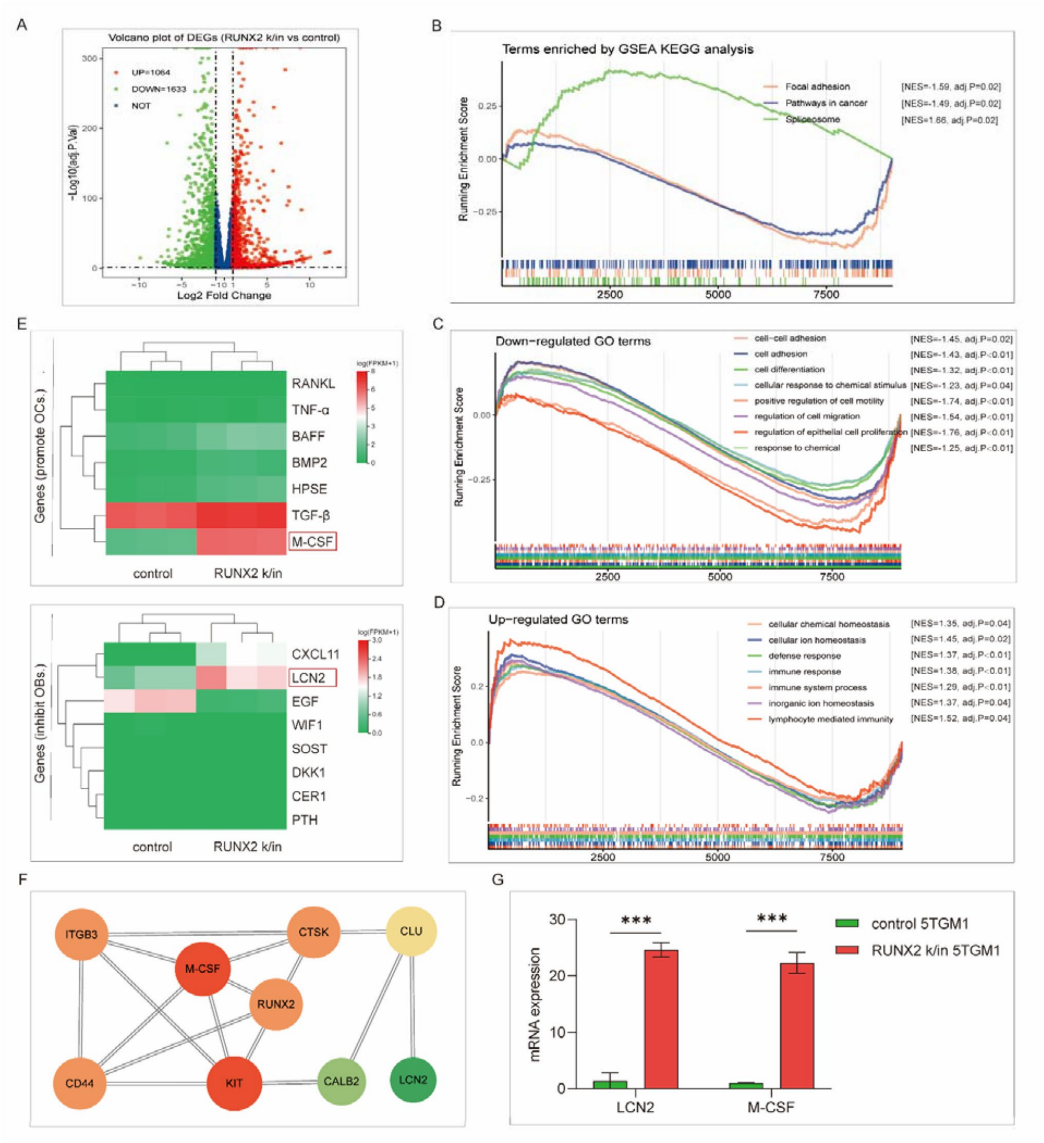
RNA-seq analysis of RUNX2 k/in and control myeloma cells. Differential expression was analyzed between RUNX2 k/5TGM1 and control 5TGM1 cells by RNA-seq. **A** Volcano plot of differentially expressed genes. **B–D** Enriched KEGG and GO terms according to GSEA. **E** Expression of genes that possibly function in osteoclast promotion or osteoblast suppression.** F** Protein‒protein interaction (PPI) analysis of RUNX2 from the RNA-seq results. **G** Expression of LCN2 and M-CSF as confirmed by qPCR

RNA-sequencing analysis was employed to explore the downstream transformation following RUNX2 upregulation with RUNX2 *k*/in and control 5TGM1 cells. A total of 1064 upregulated genes and 1633 downregulated genes were identified with an absolute log2-fold change > 1 (Fig. 5A). GSEA was performed to enrich functional terms. The upregulated Kyoto Encyclopedia of Genes and Genomes (KEGG) pathways were involved in the spliceosome, while pathways in cancer and focal adhesion were downregulated (Fig. 5B). Gene Ontology (GO) enrichment analysis showed that the downregulated terms included cell adhesion, cell differentiation, positive regulation of cell motility and regulation of cell migration, and the upregulated terms were mostly related to cellular homeostasis and the immune response (Fig. 5C–D). The enrichment results indicate novel RUNX2 pathways related to bone destruction in MM. To verify this, we analyzed the expression of genes related to osteogenesis, and LCN2 and M-CSF were upregulated following RUNX2 overexpression (Fig. 5E, G). Protein‒protein interaction (PPI) analysis supported the communication between LCN2, M-CSF, and RUNX2 (Fig. 5F). Soluble LCN2 was reported to impair osteoblast differentiation and stimulate RANKL production by osteoblasts [24]. M-CSF promotes osteoclast proliferation and acts as a costimulatory molecule with RANKL to induce osteoclast differentiation [25]. These two molecules may participate in the regulation of RUNX2 in osteogenesis, but further evidence is needed.

### RUNX2 overexpression promotes myeloma-related bone destruction in vivo

**Fig. 6:**
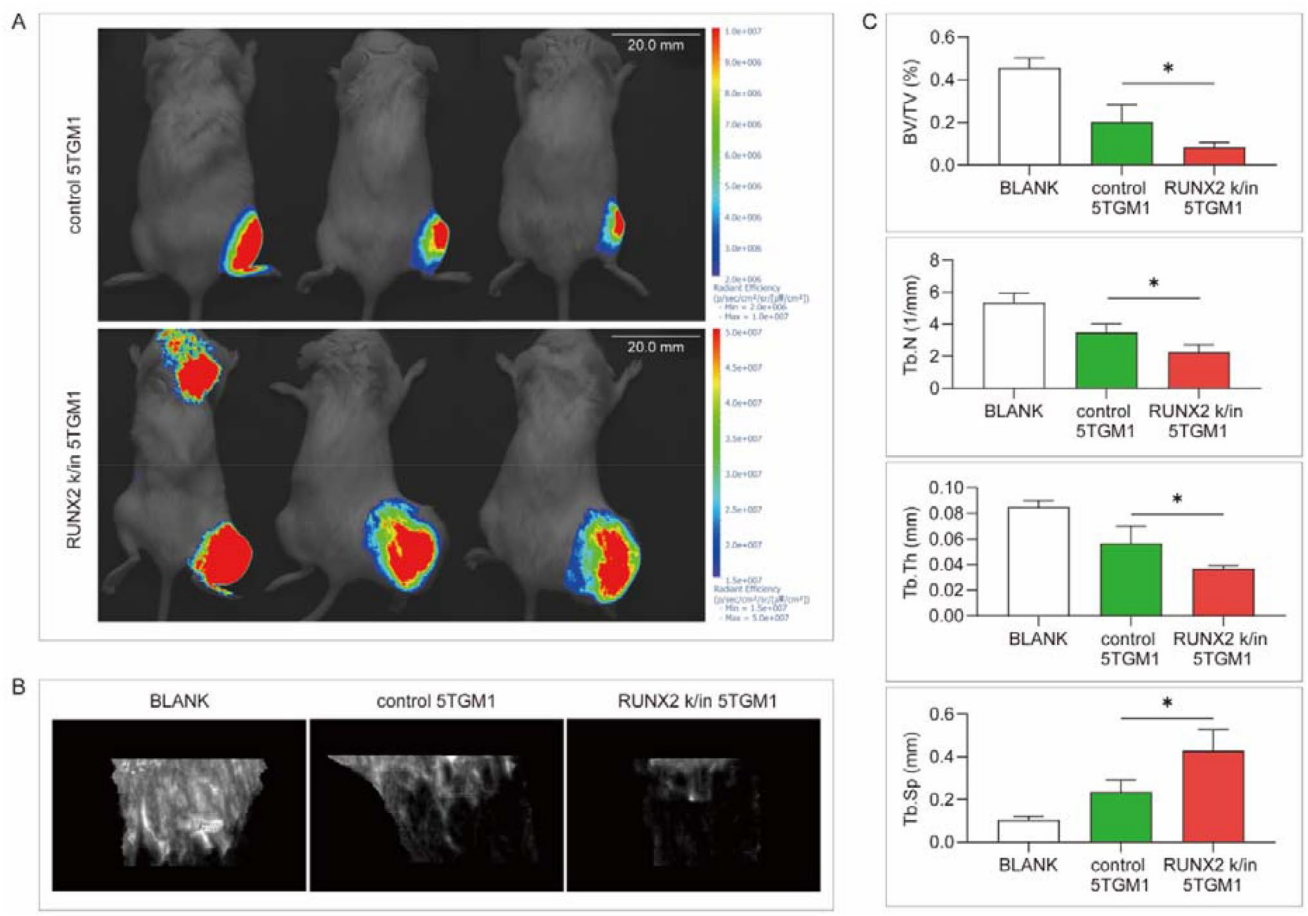
Validation of the role of RUNX2 in bone destruction in vivo. **A** Representative images of control and RUNX2 k/in 5TGM1-bearing mice 4 weeks after tibia injection. **B** Representative images of bone trabecula destruction. **C** Statistical analysis of bone volume/total volume (BV/TV), trabecular thickness (Tb.Th), trabecular number (Tb.N), and trabecular spacing (Tb.Sp) between the two groups

Myeloma-bearing mice were constructed by tibia injection with control and RUNX2 k/in 5TGM1. After 4 weeks, an in vivo imaging system (IVIS) was used to detect tumor growth and invasion, of which RUNX2 k/in group was significantly advanced compared with the control group (Fig. 6A). The affected tibias were removed after execution and assessed by micro-CT (Fig. 6B). Further statistical analysis revealed a lower ratio of bone volume/total volume, lower trabecular thickness, lower trabecular number, and higher trabecular spacing in the RUNX2 k/in group than in the control group (Fig. 6C). It has been confirmed that RUNX2 upregulation in myeloma cells aggravates bone destruction in vivo.

## Discussion

The role of RUNX2 in cancers has been explored in many studies. In human non-small cell lung cancer (NSCLC), increased expression of RUNX2 is significantly correlated with tumor size, tumor stage, and lymph node metastasis [26]. In human colon carcinoma, RUNX2 is associated with Dukes’ stage, liver metastasis, and ER*β* status, and increases in these factors are related to adverse clinical outcomes [27]. Evidence indicates RUNX2 as a poor prognostic factor in cancers, and our findings in MM support this. In an analysis of publicly available data, RUNX2 displayed elevated expression in myeloma plasma cells and was positively correlated with disease progression and unfavorable overall survival in patients.

Different from its function of promoting bone formation in osteoblasts, cancer cell-derived RUNX2 was found to have an adverse effect on cancer-related bone destruction. As reported, RUNX2 facilitates bone resorption by regulating the expression of TGFβ, MMP-9, ITGA5, or RANKL [28–31]. While most studies on RUNX2 and bone destruction focus on the influence on osteoclasts, the interaction between cancer cell-derived RUNX2 and osteoblasts is not well understood. By extracting CCM and applying it to both osteoclasts and osteoblasts, we found that RUNX2 upregulation in myeloma cells not only enhanced the promoting effect of myeloma cells on osteoclast activity but also the suppressive effect of myeloma cells on osteoblast activity and facilitated the differentiation of osteoclasts mediated by osteoblasts, ultimately aggravating bone destruction.

RNA-sequencing analysis suggested communication between RUNX2, M-CSF, ITGB3, CD44, LCN2, and CLU in RUNX2 k/in myeloma cells. Macrophage colony-stimulating factor (M-CSF) is known as a regulator of osteoclast differentiation and proliferation [32, 33]. It was reported that the combined action of M-CSF and RANKL as well as preinduction with M-CSF may promote osteoclastogenesis and enhance the resorption function of osteoclasts [34]. As studies have described, M-CSF usually works with RANKL in osteoclast differentiation [35, 36]. In our assay, RUNX2 upregulation contributed to increased expression of M-CSF but not RANKL, which indicates that M-CSF may play a role in RUNX2-induced bone destruction, but whether M-CSF acts alone needs further exploration. ITGB3 encodes integrin subunit beta 3, which was found to mediate the bone-resorbing function of osteoclast-like myeloma cells [37]. Conditional knockdown of ITGB3 revealed its involvement in osteolysis in breast cancer [38]. It has been reported in prostate cancer that integrin *α*v*β*3 and CD44 pathways function in osteoclastogenesis via a RUNX2/Smad5-signaling axis [39], which provides evidence for RUNX2-related bone destruction in MM.

Lipocalin-2 (LCN2) is a secreted glycoprotein that is abundant in aggressive subtypes of cancer, including breast, pancreas, thyroid, ovarian, colon, and bile duct cancers [40]. It was reported that LCN2 activates cAMP-mediated signaling in bone cells, while increased cAMP signaling may inhibit osteoblast differentiation by decreasing BMP pathway signaling [41, 42], which indicates the negative effect of cancer-derived LCN2 on osteogenesis. PPI networks revealed the interaction of RUNX2, CLU, and LCN2. CLU encodes secreted clusterin, which inhibits osteoblast differentiation by suppressing the ERK1/2-signaling pathway [43]; thus, we hypothesized that CLU/LCN2 signaling is involved in RUNX2-related osteoblast suppression.

In conclusion, we propose that upregulated RUNX2 promotes the suppression of osteoblast activity and enhancement of osteoclast activity by myeloma cells. Additional studies are needed to determine the role of M-CSF and LCN2 in RUNX2-related bone destruction. Further investigation of the interaction between M-CSF, LCN2, and osteoclasts or osteoblasts is needed. The study lays the foundation for further research and suggests that therapeutic inhibition of RUNX2 may protect against bone destruction by maintaining the balance between osteoblast and osteoclast activity in MM.

## Materials and methods

### Data from a public database

Gene expression profiles and clinical information of microarray datasets, including GSE24080, GSE118985, GSE77539, GSE4204, GSE31161, and GSE124435, were obtained from the Gene Expression Omnibus (GEO) database (http://www.ncbi.nlm.nih.gov/geo/). The raw count data were preprocessed with normalization and log2 transformation. Transcript sequencing data (TPM) of MM were downloaded from the MMRF-CoMMpass project on the Genomic Data Commons Data Portal (https://portal.gdc.cancer.gov/). Box plots and Kaplan–Meier survival curves were constructed with R packages “ggboxplot” and “survminer” in R Studio (version 4.2.1).

### Data from MM patients

Thirty bone marrow paraffin sections and 15 fresh bone marrow fluid samples from newly diagnosed MM patients and clinical data were collected in the First Affiliated Hospital of Sun Yat-sen University with approval from the ethics committee (No. IIT-2021-799).

Bone marrow paraffin sections were subjected to immunohistochemical staining with anti-CD138 antibody (Abcam: ab181789) and anti-RUNX2 antibody (CST: #12,556). To narrow bias brought by tumor cell density, we calculated the ratio of RUNX2/CD138 average optical density (AOD) by Image ProPlus software (version 6.0) as the relative protein level of RUNX2.

CD138-positive plasma cells were collected from fresh bone marrow fluid through immunomagnetic bead sorting (Miltenyi MACS bead isolation kit). RNA was extracted from CD138-positive plasma cells and subsequently used to assess RUNX2 expression.

### Cell lines and lentiviral transfection

The murine MM cell-line 5TGM1, preosteoblast cell-line MC3T3-E1, and preosteoclast cell-line RAW264.7 were kind gifts from Zhangxingding laboratory, Sun Yat-sen University. RUNX2 k/in and control 5TGM1 cell lines were constructed by lentiviral transfection and identified by quantitative polymerase chain reaction (qPCR) analysis and western blot analysis. Cells were cultured with medium supplemented with 10% FBS and 100 μg/mL penicillin‒streptomycin at 37 °C in 5% CO_2_.

### Preparation of CCM

RUNX2 k/in 5TGM1 cells and control 5TGM1 cells were cultured with serum-free medium at a density of 106 cells/mL for 24 h. Then, the cells were removed, and the liquid supernatant was concentrated with a 100 kD protein concentrator (Millipore: UFC9100) and filtered with a 0.22 µM pore filter at 4 °C [44]. Concentrated conditioned medium (CCM) was obtained after 10 concentration steps.

### Osteoblast and osteoclast culture

MC3T3 E1 and RAW264.7 were seeded for 24 h, and then the medium was replaced with serum-free medium (BLANK), control CCM, and RUNX2 k/in CCM. After 24 h of incubation, cell viability was detected by the CCK-8 assay (Vazyme: A311-01), apoptosis was detected with the Annexin V-APC/PI Apoptosis Kit (KeyGEN Biotech: KGA1030), and differentiation was detected by quantitative PCR according to the manufacturer’s instructions.

### Quantitative polymerase chain reaction (qPCR) analysis

qPCR was used to assess the expression of genes after total RNA was extracted and reverse transcribed using the PrimeScript cDNA Synthesis Kit (Takara: #6210). Quantitative PCR was performed using a SYBR Green Mater Mix (Vazyme: Q111-02) according to the manufacturer’s instructions. The PCR primers are listed as follows Table 1.

**Table 1:** Primers used for qPCR

Primer	Sequence
Human RUNX2	F: 5ʹ-AACCCACGAATGCACTATCCA-3 ʹ, R: 5 ʹ -CGGACATACCGAGGGACATG-3ʹ
Mouse RUNX2	F: 5ʹ-AGAGTCAGATTACAGATCCCAGG-3ʹ, R: 5ʹ-AGGAGGGGTAAGACTGGTCATA-3ʹ
Mouse OSX	F: 5ʹ-TGCGCCAGGAGTAAAGAATAG-3ʹ, R: 5ʹ-CCTGACCCGTCATCATAACTTAG -3ʹ
Mouse ALP	F: 5ʹ-GGAATACGAACRGGATGAGAAGG-3ʹ, R: 5ʹ-GGTTCCAGACATAGTGGGAATG-3ʹ
Mouse OPG	F: 5ʹ-CAATGGCTGGCTTGGTTTCATAG-3ʹ, R: 5ʹ-GGAGCTGCTGTGACATCCATAC-3ʹ
Mouse OCN	F: 5ʹ-CTCTGTCTCTCTGACCTCACAG-3ʹ, R: 5ʹ-GGAGCTGCTGTGACATCCATAC-3ʹ
Mouse RANKL	F: 5ʹ-CTCTTGGTACCACGATCGAG-3ʹ, R: 5ʹ-AAGCCCCAAAGTACGTCGCA-3ʹ
Mouse M-CSF	F: 5ʹ-GAACAGCCTGTCCCATCCATC-3ʹ, R: 5ʹ-TGAGGCCAGCTSAGCAA-3ʹ
Mouse NFATC1	F: 5ʹ-GGGTCAGTGTGACCGAAGAT -3ʹ, R: 5ʹ-AGGTGGGTGAAGACTGAAGG-3ʹ
Mouse CCND1	F: 5ʹ-GCGTACCCTGACACCAATCTC-3ʹ, R: 5ʹ-CTCCTCTTCGCACTTCTGCTC-3ʹ
Mouse CASP3	F: 5ʹ-TGGTGATGAAGGGGTCATTTATG-3ʹ, R: 5ʹ-TTCGGCTTTCCAGTCAGACTC-3ʹ

### RNA-sequencing analysis

Total RNA was extracted from control and RUNX2 k/in 5TGM1 cells. At least 1 µg of RNA from 3 biological replicates of each group was used for high-throughput RNA sequencing after quality control. Sequencing data were processed at the Beijing Genomics Institute (BGI). Transcripts per kilobase of exon model per million mapped reads (TPM) values of genes were used in the subsequent analysis. Gene Set Enrichment Analysis (GSEA) was employed to evaluate KEGG and GO term enrichment in the control and RUNX2 k/in groups with R package “clusterProfiler” in R Studio (version 4.2.1). PPI networks were constructed with 421 upregulated genes that were screened with log2-fold change > 1.5 in the STRING website (https://cn.string-db.org/) and Cytoscape software (version 3.9.1).

### Animal experiment

All procedures involving animals were approved by the Sun Yat-sen University Animal Ethics Committee (No. SYSU-IACUC-2022-000,403). C57BL/6 mice (4 weeks old) received a right tibia injection of control or RUNX2 k/in 5TGM1-GFP cells (10^6^ cells). On day 28, the animals were detected by an in vivo imaging system (IVIS) and then sacrificed. The affected tibias were assessed by microcomputed tomography (micro-CT). The 3D model of bone trabecula was constructed from 0.2 mm below the tibia platform, and trabecula parameters, including bone volume/total volume (BV/TV), trabecular thickness (Tb. Th), trabecular number (Tb. N), and trabecular spacing (Tb. Sp) were assessed.

### Statistical analysis

Statistical analysis was performed with R Studio (version 4.2.1) and GraphPad Prism software (version 9.0). The Mann–Whitney test and Student’s *t* test were used to determine significance as appropriate after the normality test. Values are presented as the mean ± standard error of the mean. A *p* value < 0.05 was considered statistically significant. All statistical tests were two-sided.

## Data Availability

The datasets generated during and/or analyzed during the current study are available from the corresponding author on reasonable request.
